# Calendula

**Published:** 1881-01

**Authors:** C. Carleton Smith

**Affiliations:** Phila.


### CALENDULA.

### *Its place in Homœopathic Therapeutics.*

BY C. CARLETON SMITH, M.D. PHILA.

THE calendula officinalis, or marsh marigold is endowed with the specific power of preventing or largely diminishing, suppuration in cases of mechanical injuries.

It is the Homœopathic topical remedy in all incised and in all lacerated wounds, and in every case where I have used it according to its indications, it has done its work promptly and satisfactorily, leaving nothing to be desired.

While arnica stands head and shoulders above every other drug as the Homœopathic remedy for injuries occasioned by blunt instruments, for bruises and contusions, calendula is the specific for lacerated wounds, no matter how severe or extensive they may be.

I may say just here that it is a most remarkable fact, that many Homœopathic physicians use arnica as the specific in all local injuries, whatever the nature of these injuries may be; and hence they blunder most woefully, and in blundering, of course, fail to cure. Arnica is always out of place in wounds or injuries where the flesh has been torn asunder and the effect of this drug in such cases is to aggravate the suffering, and in many instances to induce erysipelatous inflammation, which complicates the case and puts the life of the patient in jeopardy, especially in cases of injury involving the scalp.

Only a week ago a patient of mine aged about forty-seven, punctured the end of her left index finger, making a slight wound. The smarting it produced, caused her to apply to the wound the tincture of arnica, and instantly a sharp pain shot up the arm into the axillary region across into the chest and down into her heart producing in that organ a fixed pain which was very acute and which caused her great alarm.

Calendula never has, at least in my experience, any such untoward effect. Its application is soothing from the first moment, and invariably, provided it is used from the first, prevents suppuration, or else reduces it to a few drops at the most. Even when used late in a case, and suppuration has been going on for some time, the part healing badly, calendula will stop the suppurative process and the injury will soon heal kindly.

In severe lacerations where a large surface is involved, I employ this drug internally in an attenuated form, as well as locally, in the manner already described.

And now, in the way of illustration, let me call your attention to a few cases which have been under my care:

Case 1.—A lad, aged seven years, running with great speed while at play, came in contact with the wheel of a wagon which was being driven toward him at a rapid rate. His forehead just above the orbital region on the left side came in contact with the tire and cut out, as clean as if done with a sharp knife, a piece of flesh of an elliptic shape, an inch and a quarter in length. I saw the sufferer in course of half an hour after the accident and found the bone completely denuded. Not bebeing able to find the piece which was excised, I washed the wound carefully to divest it of all foreign matter, and then with a velvet sponge I covered the bone and the edges of the wound thoroughly with a solution of calendula made by mixing a tea-spoonful of the pure tincture with a pint of tepid water, even squeezing a few drops into the wound before closing it. I then brought the tips carefully together and united them with three sutures of white sadlers silk. At the end of two days I drew out the sutures carefully, found the parts healed thoroughly without any suppuration.

Case 2.—A young lad aged six years was playing in his father's stable. While in one of the stalls the horse that occupied it suddenly turned upon the boy, caught his upper lip between his teeth and bit out a portion about a half inch in width and the same in length the wound extending upward.

I was immediately sent for, used the calendula in the same way as in the former case closed the edges of the wound with two fine silk sutures, and applied adhesive straps to make parts more secure. The injury was healed in three days perfectly, and this, notwithstanding the fact that the patient had a crying spell in the night, waking up from a delirious sleep which had the effect of tearing out one of the sutures. In a few months after this injury the seat of it could scarcely be discovered, and both the parents declare that their son's lip is much handsomer than it was before the accident.

Case 3.—A young mechanic had the middle finger caught in the cog-wheels of a machine which he was running. Before the member could be extricated a little more than half the first joint was taken off. I saw this patient immediately after the accident; the bone was protruding and a good deal of blood was being lost. With cutting forceps, I at once removed the protruding bone, cleansed the wound carefully and after thoroughly wetting the parts with calendula drew the edges of the wound together over the end of the bone, applied adhesive plaster, and covered the whole with a linen binder, instructing the patient to keep the finger continually wet with the calendula solution. Without any further interference, the member healed nicely, a good stump was formed, and even a rudimentary nail appeared, which very greatly improved the looks of the finger.

Case 4.—This was an accident precisely similar to the case 3, and in treating it I met with the same success. In this instance, however, no signs of a new nail appeared.

Case 5.—Just before this article was written a little boy three years of age had one of his little fingers caught in the cog wheels of a wringer just back of the nail, lacerating the flesh badly but not injuring the bone. The injury was dressed by an allopathic physician, and after three weeks the case was brought to me for examination as it did not seem to be healing properly. I removed the wrappings carefully, and found suppuration going on, and the lips of the wound standing apart bathed with pus. After cleansing the surfaces brought them in apposition, applied adhesive strips, and ordered a covering for finger made of linen, and the finger to be kept constantly wet with the calendula lotion. There was no further trouble.

Remarks:—In the proving of this drug we find the following symptoms: wounds become *raw* and *inflamed*, and also become *painful* as if *beaten*, with *stinging* as if suppuration would ensue. The parts around the wound become *red*.

Great tendency to start, with great nervousness.

Restless night with constant waking, frequent drinking and uneasiness in every position.

Feels as if falling from a height.

All these symptoms are exact counterparts of those we find in patients suffering from injuries and hence the Homœopathicity of the drug in question.

